# Retinal Pathology of Pediatric Cerebral Malaria in Malawi

**DOI:** 10.1371/journal.pone.0004317

**Published:** 2009-01-29

**Authors:** Valerie A. White, Susan Lewallen, Nicholas A. V. Beare, Malcolm E. Molyneux, Terrie E. Taylor

**Affiliations:** 1 Department of Pathology and Laboratory Medicine, University of British Columbia, Vancouver, British Columbia, Canada; 2 Department of Ophthalmology and Visual Sciences, University of British Columbia, Vancouver, British Columbia, Canada; 3 British Columbia Centre for Epidemiologic and International Ophthalmology, Vancouver, British Columbia, Canada; 4 Kilimanjaro Centre for Community Ophthalmology, Kilimanjaro Christian Medical Centre, Tumaini University, Moshi, Tanzania; 5 St. Paul's Eye Unit, Royal Liverpool University Hospital, Liverpool, United Kingdom; 6 Malawi-Liverpool-Wellcome Trust Clinical Research Programme, College of Medicine, Blantyre, Malawi; 7 School of Tropical Medicine, University of Liverpool, Liverpool, United Kingdom; 8 Blantyre Malaria Project, Queen Elizabeth Central Hospital, Blantyre, Malawi; 9 College of Osteopathic Medicine, Michigan State University, East Lansing, Michigan, United States of America; WHO Initiative for Vaccine Research, Switzerland

## Abstract

**Introduction:**

The causes of coma and death in cerebral malaria remain unknown. Malarial retinopathy has been identified as an important clinical sign in the diagnosis and prognosis of cerebral malaria. As part of a larger autopsy study to determine causes of death in children with coma presenting to hospital in Blantyre, Malawi, who were fully evaluated clinically prior to death, we examined the histopathology of eyes of patients who died and underwent autopsy.

**Methodology/Principal Findings:**

Children with coma were admitted to the pediatric research ward, classified according to clinical definitions as having cerebral malaria or another cause of coma, evaluated and treated. The eyes were examined by direct and indirect ophthalmoscopy. If a child died and permission was given, a standardized autopsy was carried out. The patient was then assigned an actual cause of death according to the autopsy findings. The eyes were examined pathologically for hemorrhages, cystoid macular edema, parasite sequestration and thrombi. They were stained immunohistochemically for fibrin and CD61 to identify the components of thrombi, β-amyloid precursor protein to detect axonal damage, for fibrinogen to identify vascular leakage and for glial fibrillary acidic protein to detect gliosis. Sixty-four eyes from 64 patients were examined: 35 with cerebral malaria and 29 with comas of other causes. Cerebral malaria was distinguished by sequestration of parasitized erythrocytes, the presence and severity of retinal hemorrhages, the presence of cystoid macular edema, the occurrence and number of fibrin-platelet thrombi, the presence and amount of axonal damage and vascular leakage.

**Conclusions/Significance:**

We found significant differences in retinal histopathology between patients who died of cerebral malaria and those with other diagnoses. These histopathological findings offer insights into the etiology of malarial retinopathy and provide a pathological basis for recently described retinal capillary non-perfusion in children with malarial retinopathy. Because of the similarities between the retina and the brain it also suggests mechanisms that may contribute to coma and death in cerebral malaria.

## Introduction

In endemic countries, asymptomatic malaria parasitemia is common. Individuals with coma of a non-malarial cause may happen to be parasitemic and their illness may be wrongly identified as cerebral malaria (CM). In an autopsy study in Malawi all children with *clinically* defined CM who died without malarial retinopathy had other causes of death and an absence of cerebral sequestration.[1] In these studies the presence of malarial retinopathy during life was the best clinical predictor of sequestration of parasitized red blood cells (pRBC) in the brain. A recent analysis of 880 children with strictly defined clinical CM demonstrated that 60% had malarial retinopathy, and that this group had significantly higher case fatality rates, longer time to recovery, lower hematocrit and platelet levels, and higher lactate levels than children without retinopathy.[2] Malarial retinopathy therefore has a diagnostic and prognostic role in cerebral malaria in children.

**Figure 1:**
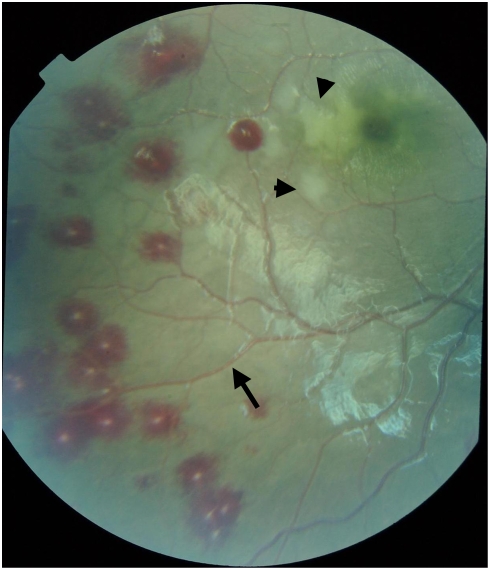
Fundus photograph displaying malarial retinopathy consisting of multiple white centered hemorrhages, macular whitening (arrowheads) and orange discoloration of vessels (arrow).

Malarial retinopathy consists of one or more of the following ocular fundus findings: hemorrhages, whitening of the retina, orange to white discoloration of the retinal vessels and papilledema (Fig. 1).[3]–[5] Lewallen et al.[6] described the histopathologic correlate of the abnormally colored retinal vessels in CM as an absence of hemoglobin in the parasitized erythrocytes that are sequestered within the retinal vasculature. Beare et al.[7] have recently demonstrated by fundus photography and fluorescin angiography that the retinal whitening in malarial retinopathy is associated with areas of non-perfusion and presumed hypoxia, and that the white retinal vessels were occluded. White et al.[8] found that the number of retinal hemorrhages correlated with number of brain hemorrhages in patients who died from CM. These previous observations all support the hypothesis that examination of the ocular fundus is useful for investigating the pathophysiology of CM as well as for diagnosis and prognosis. This is consistent with the embryology of the retina, which develops as an outpouching from the neural tube composed of neuroectodermal tissue, as is the brain.

There has not been a previous description of ophthalmic pathology from a single large series of children dying of CM. The specific aims of this manuscript are to describe the retinal pathology in patients dying of CM, to correlate pathological features with clinical findings; and to compare these with observations in children dying from fatal comas of other causes (COC).

## Materials and Methods

### Clinical Examination

Comatose children were admitted to the Research Ward of the Queen Elizabeth Central Hospital, Blantyre, Malawi. The clinical definition of cerebral malaria was *P. falciparum* parasitemia; a Blantyre coma score ≤2 and no improvement following correction of hypoglycemia, within 30 minutes of cessation of seizure activity, or within 2 hours of admission; and no evidence of meningitis on examination of cerebrospinal fluid. The clinical definition of non-malarial coma was a Blantyre coma score of ≤2 and no improvement following correction of hypoglycemia, within 30 minutes of cessation of seizure activity, or within 2 hours of admission; admitting hematocrit >15%, aparasitemic (on a minimum of 4 blood films collected every 6 hours), or parasitemic with an identifiable non-malarial cause of coma.[1]

### Ophthalmological Examination

Children's pupils were dilated and the fundi examined with direct and indirect ophthalmoscopy upon admission. The findings of an ophthalmologist or experienced clinician were recorded on standardized forms.[3], [9], [10]

### Autopsy

If the patient died, informed written consent was requested from the child's guardian in the local language, for autopsy and the use of human tissues. If this was obtained, a standardized autopsy was carried out. Representative sections were taken from multiple areas of the brain and all other organs. The eyes were removed and replaced by artificial glass eyes. The consensus classification of cause of death was made by collaborating pathologists other than the authors of this paper, and only after the complete microscopic examination of all blocks of tissue and review of microbiological cultures obtained prior to death as previously described.[1] In no case did the ocular pathology suggest a diagnosis and/or cause of death different from that of the complete autopsy.

### Gross Pathology

After fixation in formalin, the eyes were examined externally, opened horizontally in the pupil-optic nerve plane and the superior calotte removed. They were then examined under a dissecting microscope for hemorrhages and these were quantified according to the method used in the clinical examination (Grade 1:1–5 hemorrhages, Grade 2: 5–20, Grade 3: 20–50, Grade 4: >50).[10] The pupil-optic nerve tissue block was processed through graded alcohols and xylene, and embedded in paraffin wax as per standard protocol. Sections were cut at three microns, stained with hematoxylin and eosin, periodic-acid-Schiff (PAS) and used for immunohistochemistry.

### Microscopic Pathology

Hemorrhages were semi-quantitatively evaluated from 1–4 by the number of quadrants of the retina that were involved in one cross-section through the pupil-optic nerve section of the globe. The presence of cystoid macular edema (CME) was recorded when adequate sections through the macular area were obtained. The presence of fibrin thrombi was assessed as 0: absent, 1: occasional and 2: many, on both H&E and PAS stains.

### Immunopathology

All slides were stained by a routine avidin-biotin immunoperoxidase method with primary antibodies directed against β-amyloid precursor protein (APP) to assess axonal damage[11], against fibrinogen to assess breakdown of the blood-retinal barrier (BRB)[12] and against glial fibrillary acidic protein (GFAP)[13] to assess gliosis. Selected cases with large numbers of thrombi were selected for staining with an anti-fibrin antibody and for CD61 to platelets to determine the components of the thrombi.

For APP, foci of positive staining associated and not associated with hemorrhage were counted in the retina and optic nerve. The sizes of the foci were also measured with a standard micrometer and the total area expressed in square mm. Only focal, dense staining that mirrored the structure of the underlying axon was counted.

For fibrinogen, the areas of focal staining of the retina, associated and not associated, with hemorrhage were counted. The presence of fibrinogen staining of the edema spaces of CME in cases in which CME was visible were documented as positive or negative.

For GFAP, the number of cells with positively staining cytoplasm was counted in the entire area of optic nerve present on a slide and standardized to 10 square mm. In the retina GFAP antibody staining of Muller cells was recorded as 0: absent, 1: peripheral staining on one side, 2: halfway to the equator on one side, 3: to the equator on one side, and 4: present on both sides to the equator.

The study protocol was reviewed and approved by the Institutional Review Boards of the University of Malawi, University of British Columbia and Michigan State University.

### Statistics

All statistics were calculated using S-PLUS (Insightful Corporation, Seattle), version 6.2.1. Categorical values were assessed for difference with the Chi-square test or Fisher's exact test. P<0.05 was taken as the level of a significant difference between groups.

## Results

Sixty-four eyes from 64 patients were examined pathologically. This included 13 cases that were diagnosed clinically as CM, but were found on autopsy to have other causes of death. For technical reasons, not all eyes were examined for all features described.

### Parasite sequestration

**Figure 2:**
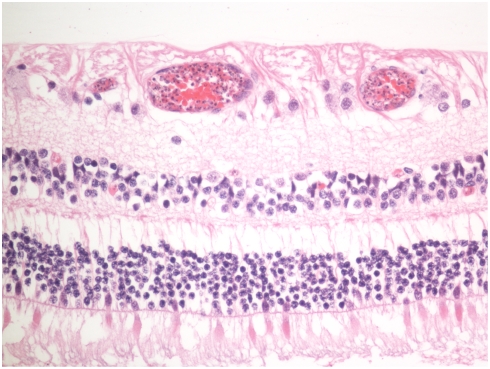
Photomicrograph of retinal vessels showing sequestration of parasitized red blood cells containing late stage parasites.

Thirty-five patients had the systemic gross and microscopic pathology typical of cerebral malaria and no other possible cause of death identified after full autopsy.[1] These cases all showed prominent sequestration of early or late stage parasites in the retinal vasculature (Fig. 2). The 29 non-malarial causes of death included acute pneumonia (5), sepsis (5), bacterial meningitis (4), hepatic failure (4), subdural/subarachnoid hemorrhage (3) and miscellaneous causes (8).

### Hemorrhages

**Figure 3:**
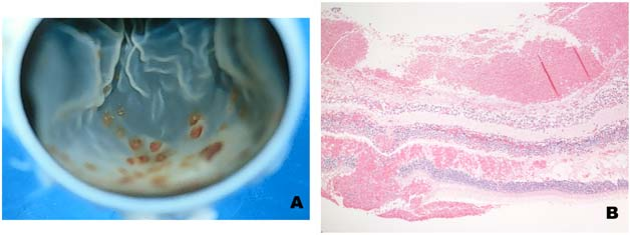
A. Representative gross photo of malarial retinopathy showing multiple white-centered hemorrhages. B. Low power photomicrograph of hemorrhages involving all layers of the retina, including beneath the internal limiting membrane as well as subretinal hemorrhage with shallow detachment.

There were significantly more hemorrhages in CM patients than in patients with non-malarial disease (Table 1)(Fig. 3A). The positive predictive value for gross retinal hemorrhages being due to CM was 93%.

**Table 1:** Master table of specific features in CM versus COC patients.

	CM	%	COC*	%	p-value
***Hemorrhages (# cases positive)***					
Clinical	17/26	65.38	4/19	21.05	0.003
Gross	25/32	78.13	2/29	6.90	<0.001
Microscopic	25/35	71.43	3/29	10.34	<0.001
***Cystoid macular edema***					
No. cases positive	17/32	53.13	1/27	3.70	<0.001
***Thrombi***					
No. cases positive	26/35	74.29	2/29	6.90	<0.001
***Axonal damage***					
No. cases positive	20/35	57.14	6/29	20.69	0.003
Mean no. of foci (SD)	1.43 (1.67)		0.34 (0.72)		0.003
Mean area (sq mm) (SD)	1.31 (1.66)		1.14 (3.57)		0.017
***Fibrinogen leakage***					
No. cases positive	11of 35	31.43	2/29	6.90	0.004
No. of foci (SD)	1.2 (2.61)		0.21 (1.11)		<0.001
***Gliosis***					
Retina, grade of severity (SD)	2.15 (1.12)		1.79 (1.01)		0.22
Optic nerve (#cells/10 sq mm) (SD)	117.22 (81.62)		126.5 (105.07)		0.86

CM = cerebral malaria.

COC = comas of other causes.

* includes 13 cases of clinically, but not pathologically, defined CM.

SD = standard deviation.

The retinal hemorrhages were usually located in the inner layers, but when numerous and large, involved all layers and were associated with subretinal hemorrhage and a shallow retinal detachment (Fig. 3B). Occasionally a vessel was surrounded by an acellular zone that in turn was surrounded by hemorrhage, similar to the appearance of a ring hemorrhage as seen in the brain.

Table 2 displays the grade of severity of retinal hemorrhages in the pathologically proven CM cases. In 6 patients, the number of hemorrhages increased by 2 or more grades between the time of the last clinical examination and the gross pathologic examination. In 7 instances, the grade of severity changed by one between the gross and microscopic exam. This is probably due to difficulty in seeing small or infrequent hemorrhages grossly against the very dark choroid, or because hemorrhages did not involve the same number of quadrants on the microscopic section as seen on gross exam. No COC cases had hemorrhages of more than Grade 1 severity (1–5 hemorrhages), except for a case with skull fractures that had numerous hemorrhages, whitening and papilledema. This included the 13 cases with clinically, but not pathologically, defined CM. No COC cases had vessel abnormalities.

**Table 2:** Severity of hemorrhages in CM cases based on clinical grading scale*.

	0	1	2	3	4	NA
Clinical	9	10	2	5	0	9
Gross	7	9	2	11	3	3
Micro	10	11	2	2	10	0

* 0 = none.

1 = 1–5.

2 = 5–20.

3 = 20–50.

4 = 50+.

CM = cerebral malaria.

NA = data not available.

### CME

**Figure 4:**
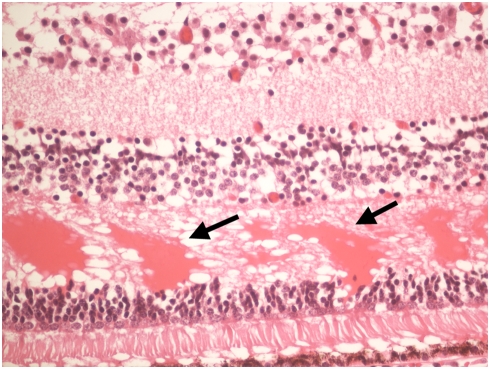
Photomicrograph of cystoid macular edema in the outer plexiform layer (arrows).

CME was significantly more common in the CM group than the non-CM group (Table 1)(Fig. 4).

### Thrombi

**Figure 5:**
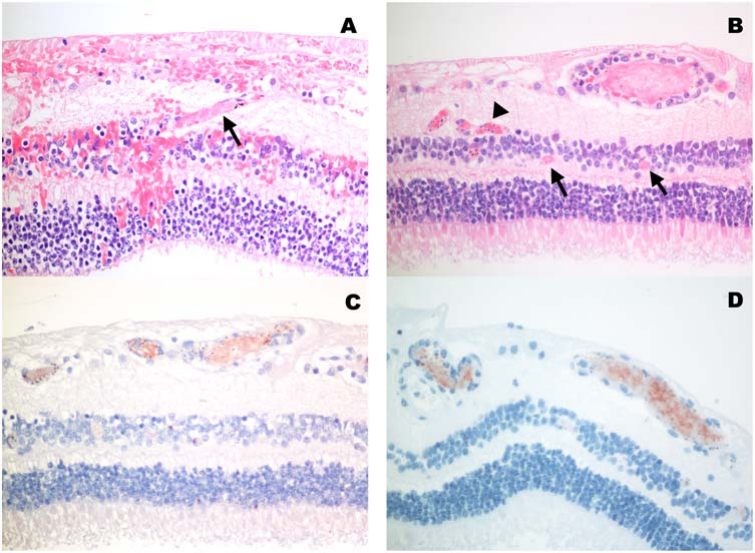
A. Photomicrograph of retinal hemorrhage with a central vessel containing a thrombus sectioned obliquely (arrow). B. Thrombus in a larger vessel without surrounding hemorrhage. Note that some of the surrounding capillary-sized vessels contain thrombi (arrows) while others do not (arrowhead). C&D. Immunohistochemical staining for fibrin (C), and CD61 to identify platelets (D). (C: Anti-fibrin, hematoxylin counterstain; D: Anti-CD61, hematoxylin counterstain.)

Thrombi were frequently seen in the retinal vessels in cerebral malaria (Fig. 5A,B) with very few in non-CM cases (Table 1), the difference being highly significant. In a semi-quantitative assessment, 16 cases of CM had “many” thrombi, compared to only one non-CM case, a patient with skull fractures, Thrombi were sometimes associated with hemorrhage, but were often seen without surrounding hemorrhage. Frequently thrombi only occluded a portion of the vessel lumen if the vessel was larger than a capillary. On light microscopy thrombi appeared to be composed of fibrin only with occasional pigment granules, but on immunohistochemistry the thrombi stained for both fibrin and platelets (Fig. 5C,D)

### Axonal damage

**Figure 6:**
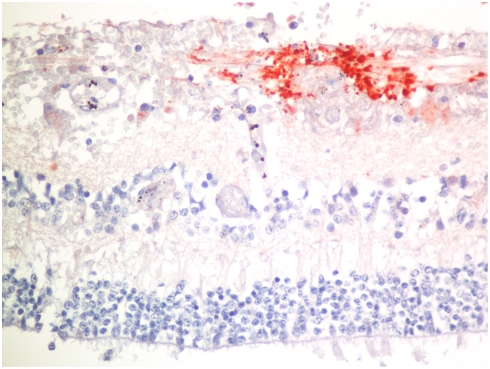
Photomicrograph of immunohistochemical staining for β-APP in nerve fiber layer. (Anti-β-APP, hematoxylin counterstain.)

The number of cases positive for any APP staining in the CM group was significantly higher than the number in the COC group (Table 1). The staining was located in the nerve fiber layer of the retina and could be present with or without hemorrhage. Most of the foci were small and punctate, but larger collections were occasionally visible (Fig. 6). The mean number of foci of staining and the average area of staining was also significantly greater in CM. Most of the staining in the COC group was accounted for by one case of non-accidental trauma where there were 18 mm^2^ of staining of axons, all in the anterior optic nerve, a feature which has previously been observed in this situation, but which we did not observe in any case of CM.[14]

### Breakdown of BRB

**Figure 7:**
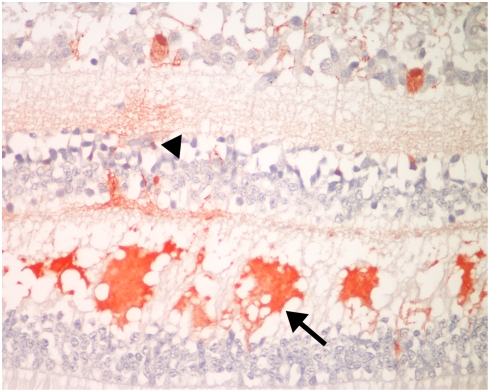
Photomicrograph of immunohistochemical staining for fibrinogen surrounding a small vessel (arrowhead) and in the spaces of cystoid macular edema (arrow). (Anti-fibrinogen, hematoxylin counterstain.)

In CM staining for fibrinogen was present around all vessels with hemorrhage, but was also present around blood vessels that were not associated with hemorrhage (Fig. 7). The number of cases positive for fibrinogen staining not associated with hemorrhage and the total number of foci of fibrinogen staining was significantly greater in the CM than the COC cases (Table 1). All cases with CME visible in the sections showed staining of the cystic spaces for fibrinogen (Fig. 7).

### Gliosis

**Figure 8:**
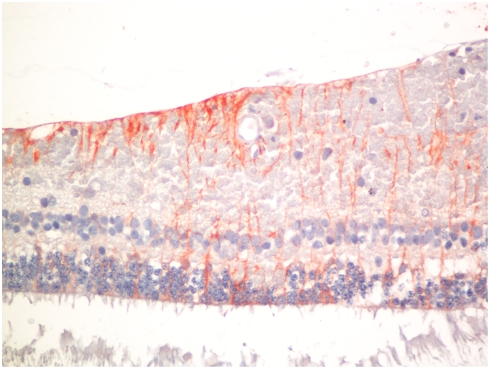
Photomicrograph of immunohistochemical staining for glial fibrillary acidic protein in retinal Muller cells. (Anti-GFAP, hematoxylin counterstain.)

Table 1 shows the mean scores for staining of Muller cells in the retina (Fig. 8) and astrocytes in the optic nerve. There was no difference between the CM and COC cases in these two measures of gliosis.

## Discussion

In this study we have documented the gross and microscopic pathology of the retina in the eyes of a large series of Malawian children dying from illnesses causing coma, including cerebral malaria and a variety of other conditions. We have attempted to relate the pathological findings to ophthalmological features observed during the illness. The extent of all pathological features, except gliosis, differed significantly between the malaria and non-malaria cases.

Retinal hemorrhages are frequent in cerebral malaria, being reported in 40–60% of cases.[9], [15]–[17] In the group of fatal cases described in this paper, the prevalence was approximately 70%. While retinal hemorrhages can occur in other conditions causing coma, in this malaria endemic setting, the presence of retinal hemorrhages, particularly when white-centered, is highly suggestive of a diagnosis of CM. While the presence/absence of hemorrhages has not been found to be predictive of outcome in some studies, Beare et al.[17] found that a large number of hemorrhages was predictive of death, particularly when a large increase was observed over serial examinations. In 6/35 cases of CM in this study the number of hemorrhages increased by 2 grades between clinical and pathological examination. Our investigation also showed that non-CM patients had none or only a few hemorrhages (Grade 1 severity, <5 hemorrhages). Hemorrhages are the easiest of the fundoscopic findings of malarial retinopathy for inexperienced examiners to identify, and they are readily seen with a direct ophthalmoscope and dilation of the pupil. They should be looked for in all cases of suspected CM.

Retinal hemorrhages are similar histopathologically to ring hemorrhages in the brain in CM. They usually occur in the inner and middle layers of the retina, but when more frequent and larger, involve all retinal layers and can be associated with subretinal hemorrhage and shallow retinal detachment.

The presence of thrombi has been described previously in cases of cerebral malaria.[18] This observation is supported by the clinical ophthalmoscopic finding of white-centered hemorrhages, which are known to be associated with a thrombus in the small vessel at the centre of the hemorrhage and which may be seen in a variety of conditions.[19] The data on the composition of thrombi are conflicting: Grau et al.[20] found platelet specific glycoprotein GPIIb-IIIa by immunohistochemical staining on frozen sections of brains from six of the CM cases included in this study, postulating that the thrombi are composed of both fibrin and platelets. By contrast Macpherson et al.[21] reported the presence of intravascular fibrin deposition in a mean of 10% (0–44%) of vessels in an electron micrographic study of brain specimens and other organs from adult patients dying of CM and severe non-cerebral malaria in Thailand. They did not see platelets and suggested that thrombosis played no role in the etiology of CM. Similarly, Pongponratn et al.[22] described only occasional clumps of fibrin in cerebral vessels, but no fibrin-platelet thrombi, in their electron micrographic study of adults from Thailand and Vietnam. Our study confirms that thrombi are more frequent in pediatric CM patients than in COC and that the thrombi are composed of both fibrin and platelets confirming the findings of Grau et al.[20]. This corroborates and may be responsible for the low circulating platelet counts characteristic of malaria and also reported by Lewallen et al.[2] in children with malaria retinopathy. That thrombi are frequently seen histopathologically in brain and retinal vessels without associated hemorrhage suggests that the thrombus occurs prior to hemorrhage or may not always produce a hemorrhage. As the thrombi do not always completely occlude the vessel, vascular flow may sometimes be compromised without being completely obstructed.

Retinal thrombi are present in diseases that produce a fundoscopic retinal appearance called Purtscher retinopathy, similar in some respects to the retinal whitening in malarial retinopathy.[23] These include diseases in which clotting is abnormal, such as hemolytic-uremic syndrome[24], thrombotic thrombocytopenic purpura[25], and pancreatitis[26], [27]. In some of these cases fluorescein angiography has revealed areas of retinal non-perfusion and pathologic examination of cases of Purtscher retinopathy has revealed occlusion of the small vessels by thrombi. Beare et al.[7] have recently shown that areas of non-perfusion on fluorescein angiography of Malawian patients with CM correspond to the areas of retinal whitening. We propose that thrombi in CM contribute to ischemia and subsequent hypoxia in the tissue that plays an important role in causing the retinal whitening, which results from loss of retinal transparency. It has been postulated that whitening develops when the retina reaches a threshold of ischemia and/or hypoxia and intra-cellular edema occurs.[28]

The concept of cerebral edema in CM is not new, but evidence has been lacking. At gross examination, the brain in fatal CM is commonly swollen, but uncal herniation is rare and there is limited histopathological evidence of cerebral edema.[29] We used immunohistochemical staining of fibrinogen, a large protein that is normally confined to the intravascular compartment, to demonstrate breakdown of the BRB in our cases[12]. As expected, we found extravascular fibrinogen in areas of hemorrhage, but it was also present around vessels that were not involved in a hemorrhage and in eyes that had none or only a few hemorrhages. Brown et al.[30] studied eight Malawian children who died from CM for evidence of blood-brain barrier dysfunction and found a decrease in cell junction proteins on endothelial cells in the vicinity of sequestered pRBC, but no leakage of fibrinogen into the surrounding brain parenchyma. Conversely, some of the same investigators found leakage of fibrinogen into the perivascular parenchyma in Vietnamese adults who died of CM. In both instances they found staining of perivascular macrophages for macrophage scavenger receptor and sialoadhesin, implying that these cells had been activated by exposure to an intravascular protein.[31] They postulated that there is widespread upregulation of the cerebral microvascular endothelium in CM with focal breakdown of the blood-brain barrier, in the absence of macroscopic edema. Our findings in the retina in fatal malaria were similar.

In support of the breakdown of the BRB is our frequent histologic finding of CME and of staining of the fluid within the intraretinal cystic spaces for fibrinogen in CM patients. In CME, fluid accumulates between the Muller cell and photoreceptor cell processes in the outer plexiform layer of the macular area of the retina producing an appearance of multiple small cystic intraretinal spaces on ophthalmoscopic exam. It can occur after intraocular surgery, in diabetics, retinal vein occlusion and ocular inflammatory diseases, all conditions where vascular integrity is impaired.[32], [33] This finding was seen on ophthalmoscopy in only a few patients, but it was identified in many more pathologically. This may be because the presence of macular whitening impairs the assessment of CME, or that indirect ophthalmoscopy is a relatively insensitive technique for detecting microscopic edema or because the development of CME is an agonal event occurring between the time of ophthalmoscopy and death.

Axonal damage as demonstrated by immunohistochemical staining for APP is seen in numerous CNS diseases.[34] It is a sensitive marker of axonal damage, becoming visible within 1 to 3 hours of injury and persisting for months.[11] Medana et al.[35] identified several patterns of APP staining in the brains of Vietnamese adults dying of CM. When present in the retina, it was visible in the nerve fiber layer or pre-laminar optic nerve. There were significant differences between the CM and non-malarial cases in both the number of cases that had APP staining and the amount of staining that was present. Not all APP staining was associated with local hemorrhage, although we cannot rule out that hemorrhage was present in adjacent sections in some instances. Axonal damage may also result from hypoxia due to complete or partial vascular occlusion by sequestered parasites and/or fibrin-platelet thrombi.

In this study, gliosis was the only finding that was not different in CM and non-CM cases in both the retina and optic nerve. We postulate that this is because the causes of death in the non-malarial cases would also lead to gliosis, although not necessarily by the same mechanism as in CM.

### Summary

We have described the histopathological correlates of malarial retinopathy. We found significant differences in retinal pathology between patients who died of CM and those with other diagnoses. CM was distinguished by the presence and severity of retinal hemorrhages, the presence of cystoid macular edema, the occurrence and number of fibrin-platelet thrombi, and the presence and amount of axonal damage and vascular leakage. The retinal pathology we have described offers insights into the etiology of malarial retinopathy and, because of the similarities between the retina and the brain, suggests mechanisms that may contribute to coma and death in cerebral malaria.
